# Role of Point-of-Care Ultrasound in Diagnosing Cysticercosis

**DOI:** 10.7759/cureus.17013

**Published:** 2021-08-08

**Authors:** Sasikumar Mahalingam, Gunaseelan R, Shivani Karn, Shirshendu Dhar, Suruthi Purushothaman

**Affiliations:** 1 Emergency Medicine, Jawaharlal Institute of Postgraduate Medical Education and Research, Puducherry, IND; 2 Dermatology, Rangaraya Medical College, Kakinada, IND

**Keywords:** myocutaneous cysticercosis, neurocysticercosis, point-of-care-ultrasound, subcutaneous swelling, lipoma

## Abstract

Soft-tissue swelling is common in clinical practice and few common causes are lipoma, sebaceous cyst, abscess, etc. Though rare, myocutaneous cysticercosis can also be a differential. Point-of-care ultrasound (POCUS) can help diagnose myocutaneous cysticercosis, which can predict neurocysticercosis among patients presenting with headaches and seizures. Myocutaneous cysticercosis is often seen as a cystic lesion with eccentric hyperechoic foci (scolex) in ultrasound. Here, we describe a case of multiple swellings in the neck and forearm associated with headache, which turned out to be myocutaneous cysticercosis and neurocysticercosis, diagnosed with the help of ultrasound.

## Introduction

Cysticercosis is a parasitic disease caused by Taenia solium and is commonly seen in developing countries. Data on the global prevalence of neurocysticercosis are limited. Community-based studies of epilepsy in India have reported variable prevalence rates, 5−10/1,000 population. Cysticercosis is highly prevalent in the northern states of Bihar, Orissa, Uttar Pradesh, and Punjab, where the prevalence can be still higher. Neurocysticercosis accounts for anywhere between 8.7% and 50% of patients presenting with recent onset of a seizure. It is transmitted to humans owing to poor hygiene and improper sanitation. Though endemic in several developing countries, several cases have been diagnosed in developed countries due to globalization. It commonly involves the central nervous system, followed by eyes, skin, and soft tissues. It can affect other areas like the spinal cord, orbit, heart, lungs, peritoneum, breast, skeletal muscle, oral cavity, tongue, etc. The signs and symptoms depend on the location of the parasite and the immunological response of the host to the parasite. Neurocysticercosis is the major cause of acquired epilepsy worldwide. Though muscular cysticercosis remains asymptomatic for a long time, neurocysticercosis presents many signs and symptoms. Myocutaneous cysticercosis can be an early predictor of central nervous involvement of cysticercosis. Here, we report a case of myocutaneous cysticercosis diagnosed with the point-of-care ultrasound (POCUS), which helped us predict neurocysticercosis in our patient.

## Case presentation

**Figure 1:**
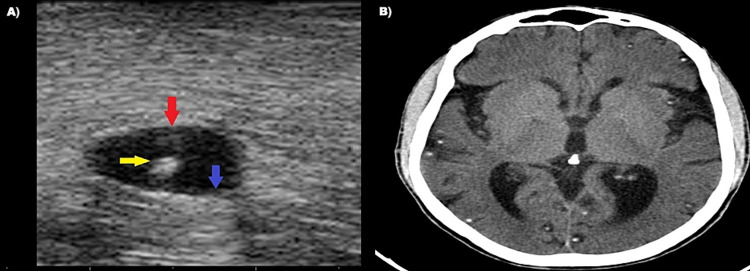
(A) Point-of-care soft tissue ultrasound of neck swellings showing cystic lesion (red arrow) with a hyperechoic focus (scolex - yellow arrow) and internal debris (blue arrow) suggesting soft tissue cysticercosis. (B) Computed tomography of the brain showing multiple cysticercoid granuloma with perilesional edema and calcification suggesting neurocysticercosis.

A 50-year-old male presented to our emergency department with complaints of multiple swellings involving the neck and forearm regions for one year and occasional on and off headaches for one year, which had aggravated for the past one week. No other significant complaints were present. Vitals and systemic examination were normal. Local examination of neck and forearm showed multiple small swellings, each measuring approximately 1*2 cm in size. We did point of care soft tissue ultrasound in the neck and forearm, which showed multiple small cystic lesions with each measuring 1* 2 cm (Figure 1A). These cystic lesions had eccentric hyperechoic foci within and minimal debris suggesting myocutaneous cysticercosis. These ultrasound features helped us predict neurocysticercosis as the diagnosis before taking computed tomography (CT) of the brain. We confirmed the diagnosis with CT brain, which showed multiple cystic lesions in the brain, with some showing perilesional edema and some showing calcification (Figure 1B). The patient was started on prophylactic antiepileptics and steroids (titrated over one month). Antihelminthic agents were avoided due to the risk of reaction around the extensively distributed cysts in the brain and myocutaneous tissue. On follow-up, the patient was symptomatically better.

## Discussion

Taenia solium is a human parasite that causes two distinct diseases in man: taeniasis, the infestation of the small intestine with the adult tapeworm, and cysticercosis, the infection of the organs and tissues with its larvae. Cysticercosis is an infection of both humans and pigs with the larval stages of Taenia solium. Humans are both definitive host and intermediate host, whereas pigs are intermediate hosts. This infection is caused by the ingestion of shredded eggs in the feces of a human tapeworm carrier. These eggs do not require a developmental period outside the host; hence they are infectious immediately after shedding when ingested by humans and pigs. Ingestion of contaminated food/water with feces containing eggs/proglottids, person to person spread, and feco-oral transmission are the modes of human exposure. Oncospheres hatches from eggs/ gravid proglottids in the intestine and start invading the intestinal wall, enters the bloodstream, leading to dissemination where they mature into cysticerci with scolex [1].

Cysticercosis can be seen in both vegetarians and non-vegetarians. This finding indicates generalized exposure to eggs through contaminated fruits or raw vegetables. The clinical onset occurs months to years after infestation, and the most frequent manifestation is epilepsy. It can affect any organ or tissue in the body, namely brain, spinal cord, eyes, orbit, heart, lungs, peritoneum, breast, skeletal muscle, oral cavity, tongue, but commonly affects the subcutaneous tissue, central nervous system, eyes, and skeletal muscle [2]. The clinical features depend on the organ involved, the number of cysts, and the host reaction [3]. Clinical features are often due to cystic degeneration and the release of antigenic materials. Though it affects several organs, its morbidity is related to the central nervous system and ocular involvement. Cysticercosis, especially neurocysticercosis, is a significant public health problem in India. Diagnosing subcutaneous cysticercosis is essential as it can help in the early diagnosis of neurocysticercosis, and prevention of seizure [1].

Muscle or subcutaneous tissue involvement is the most common site of extraneural cysticercosis. Muscular cysticercosis is often associated with neurocysticercosis. Cutaneous cysticercosis presents as multiple mobile, firm, round, subcutaneous papulonodular swellings, which are primarily asymptomatic. Myocutaneous cysticercosis is most often painless swelling, which goes unnoticed as most of them are asymptomatic. Rarely, myocutaneous cysticercosis can manifest with muscular pain, weakness, or pseudohypertrophy. Due to heavy infection of the skeletal muscles, muscular pseudohypertrophy can occur, which gives a typical “herculean appearance” to the patient [4]. Muscular cysticercosis is often classified as Myalgic type, pseudotumor/abscess type, or pseudohypertrophy type. The viable cyst usually induces no or minimum immune response, but the dying cysticercus induces inflammation which can be painful and cause discomfort. They are commonly seen in the upper extremities and the trunk. The diagnosis can be made by ultrasonographic examination of the nodule in situ or histopathologic analysis of an excised nodule that showed a cyst containing the cysticercus larvae. Intramuscular cysts often calcify and can thus be detected by radiographic examination. Cutaneous cysticerci do not carry any risks to the patient’s health, but they are often a pointer to the involvement of internal organs, like the brain, but can also be an isolated finding [5].

Myocutaneous cysticercosis can be misdiagnosed as lipoma, abscess, pyomyositis, tuberculous lymphadenitis, epidermoid cyst, ganglion, neurofibromas, or fat necrosis. Point-of-care soft tissue ultrasound of myocutaneous cysticercosis is often seen as a cystic lesion with internal debris, an eccentric hyperechoic focus (scolex) [6]. Other USG features can be abscess surrounding the cysticercus lesion with scolex within [7]. These features can help in differentiating soft tissue cysticercosis from other conditions. The X-ray can show soft tissue calcification. The differential diagnosis of soft tissue calcification is pretty narrow. Hence, the diffuse “millet seed” appearance of the calcification seen with cysticercosis is fairly pathognomonic for cysticercosis. The definitive diagnosis of subcutaneous cysticercosis is made by biopsy and histologic examination. The specimen is a fluid-filled opaque cyst that contains a single solid, white sphere - the scolex [8].

Neurocysticercosis is the commonest form reported, with the brain parenchyma being the commonest site, followed by meninges, ventricles, eye, and spinal cord. Neurocysticercosis can present with seizures, headache, nausea, and vomiting from elevated intracranial pressure, focal neurological deficits, psychological disturbances, and dementia. Seizures are more often focal than generalized. Rare central nervous manifestations include optic nerve involvement, dorsal midbrain syndrome, isolated bilateral ptosis, papillitis, cerebral hemorrhage, painful cervical radiculopathy, and paraplegia due to intramedullary cyst in the spinal cord [9]. Common cerebrospinal fluid findings are low glucose, elevated protein, and eosinophilic pleocytosis. Neurocysticercosis can be asymptomatic in up to 50% of patients if there is a lack of host inflammatory response as seen in immunocompromised patients [5]. Neurocysticercosis is diagnosed based on the distinctive appearance of CT scans, namely, cystic structures with centralized calcifications. MRI is considered the best neuroimaging tool for radiological diagnosis of neurocysticercosis with the advantage of being able to differentiate the stages of the parasite [4].

Treatment options available are conservative, medical, or surgical. A single asymptomatic lesion can be treated conservatively. Myocutaneous cysticercosis with abscesses can be treated surgically. Multiple active cystic lesions can be managed medically using steroids (prednisolone [1mg/kg/day] or dexamethasone {0.1mg/kg/day]), antihelminthic agents (albendazole [10-15mg/kg/day in two divided doses], praziquantel [50mg/kg/day in three divided doses]) and antiepileptics if needed. Dexamethasone has been shown to decrease plasma levels of praziquantel by 50%. Hence, this combination has to be avoided [10]. Antihelminthic therapy and steroids do not reduce the development of calcification and the risk of chronic epilepsy. These are the widely accepted regimen for systemic or neurological or multiple cutaneous cysticercoses. The treatment duration is usually three weeks. An important precaution to be kept in mind before instituting antihelminthic drugs is orbital/ocular cysticercosis should be ruled out. A single antiparasitic agent (albendazole) can be used for fewer cystic lesions, where both albendazole and praziquantel are needed for multiple cystic lesions. Albendazole is more effective and less expensive as compared to praziquantel. A problem with antihelminthic therapy is that it causes accumulation of inflammatory cells around cysticerci, leading to clinical deterioration, which is more dangerous in patients with a heavy parasitic load. Hence, systemic corticosteroids should be started at least one or two days before the antiparasitic treatment, continued, and gradually tapered along with the treatment [7]. Steroids can help to control the edema and intracranial hypertension that may occur following therapy. Rarely, this may be fatal in heavy infections, despite the administration of corticosteroids. After three weeks of treatment, the patient can be reassessed for resolution of clinical symptoms and resolution of soft tissue lesions by ultrasound.

## Conclusions

Myocutaneous cysticercosis should be considered one of the differential diagnoses in any patient presenting with cutaneous or subcutaneous swelling. POCUS can be used to differentiate myocutaneous cysticercosis from other subcutaneous swellings. Skin lesions can assist as a marker in the diagnosis of asymptomatic neurocysticercosis. Skin lesions can be a predictor of systemic cysticercosis among patients presenting with systemic symptoms like a visual disturbance in ocular cysticercosis, rarely weakness in spinal cord cysticercosis, etc. Physicians also must be aware of the importance of diagnosing cysticercus subcutaneous nodule by clinical examination and POCUS, as it may help in diagnosing neurocysticercosis, which may be asymptomatic for a long time. The best way to control cysticercosis would be to prevent infestation by means of education about personal hygiene and the improvement of public sanitation.
